# Large-scale systematic analysis of exposure to multiple cancer risk factors and the associations between exposure patterns and cancer incidence

**DOI:** 10.1038/s41598-021-81463-6

**Published:** 2021-01-27

**Authors:** Julia Steinberg, Sarsha Yap, David Goldsbury, Visalini Nair-Shalliker, Emily Banks, Karen Canfell, Dianne L. O’Connell

**Affiliations:** 1 Cancer Research Division, Cancer Council NSW, Sydney, NSW Australia; 2 Sydney School of Public Health, University of Sydney, Sydney, NSW Australia; 3 National Centre for Epidemiology and Population Health, Australian National University, Canberra, Australia; 4 Prince of Wales Clinical School, UNSW Medicine, Sydney, NSW Australia; 5 School of Medicine and Public Health, University of Newcastle, Newcastle, NSW Australia

**Keywords:** Cancer epidemiology, Risk factors

## Abstract

Exposures to cancer risk factors such as smoking and alcohol are not mutually independent. We aimed to identify risk factor exposure patterns and their associations with sociodemographic characteristics and cancer incidence. We considered 120,771 female and, separately, 100,891 male participants of the Australian prospective cohort 45 and Up Study. Factor analysis grouped 36 self-reported variables into 8 combined factors each for females (largely representing ‘smoking’, ‘alcohol’, ‘vigorous exercise’, ‘age at childbirth’, ‘Menopausal Hormone Therapy’, ‘parity and breastfeeding’, ‘standing/sitting’, ‘fruit and vegetables’) and males (largely representing ‘smoking’, ‘alcohol’, ‘vigorous exercise’, ‘urology and health’, ‘moderate exercise’, ‘standing/sitting’, ‘fruit and vegetables’, ‘meat and BMI’). Associations with cancer incidence were investigated using multivariable logistic regression (4–8 years follow-up: 6193 females, 8749 males diagnosed with cancer). After multiple-testing correction, we identified 10 associations between combined factors and cancer incidence for females and 6 for males, of which 14 represent well-known relationships (e.g. bowel cancer: females ‘smoking’ factor Odds Ratio (OR) 1.16 (95% Confidence Interval (CI) 1.08–1.25), males ‘smoking’ factor OR 1.15 (95% CI 1.07–1.23)), providing evidence for the validity of this approach. The catalogue of associations between exposure patterns, sociodemographic characteristics, and cancer incidence can help inform design of future studies and targeted prevention programmes.

## Introduction

Lifestyle factors such as smoking, alcohol intake, diet and physical activity play a major role in the aetiology of different cancers^1^. However, exposures to these lifestyle factors are not independent of each other—for example, there are known links between exposures to raised Body Mass Index (BMI), lack of exercise, and poor diet, and thus it is unlikely that these exposures will have isolated effects on health^2^. It is therefore important to establish the relationships between different risk factors, identify exposure patterns and their sociodemographic associations, and examine the joint associations of exposure patterns with cancer incidence, so that cancer risks can be better understood and addressed.

Factor analysis is a statistical approach that condenses multiple individual lifestyle risk variables into a smaller set of so-called “latent factors” (labelled “combined factors” in this paper) which capture variation in individual lifestyle risk variables. A number of previous cancer risk studies have applied factor analysis to diet and nutrition variables (e.g.^3–6^), and separately, to reproductive variables (e.g.^7^). Factor analysis is related to latent profile models, which have also been applied to lifestyle information (e.g.^8^). The main difference is that latent profile models assume latent variables are categorical (e.g. present or absent) and correspondingly seek to divide individuals into discrete separate groups based on their lifestyle (e.g. “High risk” versus “Low risk”). By contrast, factor analysis considers continuous latent variables and returns a continuous score for each individual and each latent factor (e.g. continuous risk behaviour level), retaining more granular risk information which facilitates the later examination of dose–response relationships.

In this study, we jointly examined 36 different lifestyle factors (including smoking, alcohol intake, diet, BMI, physical activity, sedentary behaviour, reproductive history) in a large Australian cohort. We investigated relationships between these risk variables and applied factor analysis to identify “combined factors” reflecting exposure patterns. To understand the variation in these factors across different population groups, we examined the associations between the combined factors and ancestry, health and socioeconomic characteristics.

We then systematically tested the associations between the combined factors and the incidence of several major cancers (lung, bowel, breast, prostate cancer, and melanoma) as well as all invasive cancers combined. Finally, we tested for possible interaction effects between the combined factors on cancer incidence.

## Methods

### Data sources

We used data from The Sax Institute’s 45 and Up Study, a longitudinal study of 267,153 Australian residents, described in detail elsewhere^9^. Briefly, a random sample of New South Wales (NSW) residents aged ≥ 45 years from the Medicare Australia enrolment database held by Services Australia (formerly the Department of Human Services) was invited to participate in the study. The database provides near complete coverage of the population. Individuals aged 80 and over, as well as those living in regional and remote areas, were oversampled by a factor of two during recruitment. About 18% of those invited participated, with participants comprising about 11% of the NSW population aged 45 years and over. Participants completed a baseline questionnaire between January 2006 and December 2009 (78% completed the baseline questionnaire in 2008). All participants gave written informed consent for follow-up and linkage of their information to routine health databases.

The 45 and Up Study data include: (1) reimbursements for subsidised outpatient and medical services and some in-hospital procedures covered by the Medicare Benefits Schedule (MBS); (2) inpatient care in public and private hospitals in NSW from the Admitted Patient Data Collection (APDC); (3) emergency department presentation records from the NSW Emergency Department Data Collection (EDDC); (4) cancer diagnoses (excluding non-melanoma skin cancer) from the population-wide NSW Cancer Registry (NSWCR); and (5) death records from the NSW Registry of Births, Deaths and Marriages (RBDM). Individual records were linked to the health database (1) by the Sax Institute using a unique identifier that was provided to Services Australia. NSW Health data for (2) to (5) were provided by the NSW Ministry of Health and Cancer Institute NSW, and individual records were probabilistically linked by the Centre for Health Record Linkage in NSW (CHeReL, http://www.cherel.org.au/) using a best practice approach to linkage while preserving privacy^10^. The NSWCR has high standards of data completeness and quality, and the data are accepted by the International Agency for Research on Cancer for publication in Cancer Incidence in Five Continents^11^.

The study questionnaire is available at https://www.saxinstitute.org.au/our-work/45-up-study/questionnaires/. A participant’s gender (coded as male or female) used in the analysis was obtained at baseline from the Medicare Australia enrolment database or information from the participant that the incorrect baseline questionnaire had been sent. We note that the usual gender terminology refers to “women” or “men”, but have used the terms contained in the Medicare Australia data and the 45 and Up Study data (“male” or “female”).

The conduct of the 45 and Up Study was approved by the University of New South Wales Human Research Ethics Committee. The work in this paper was approved as part of a larger research programme by the NSW Population and Health Services Research Ethics Committee (approval number 2014/08/551), and was performed in accordance with all relevant guidelines and regulations.

### Study sample for correlations between risk variables, identification of combined factors

**Figure 1:**
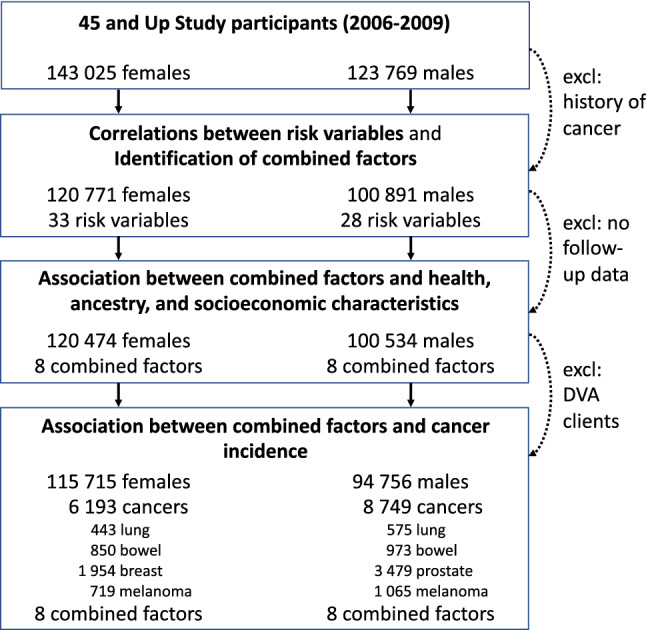
Overview of the study, included participants and incident cancers. *excl* excluded; *DVA* Australian Government Department of Veterans’ Affairs (clients excluded due to incomplete data capture).

We excluded 45 participants with probable linkage errors (e.g. multiple hospital admissions after date of death), and those with cancer history at baseline (self-reported or in cancer registry, excluding non-melanoma skin cancer), retaining 120,771 females and 100,891 males (Fig. 1). We considered 33 cancer risk variables for females and 28 for males (definitions and summary statistics see Table 1). The risk exposure information for each participant was collected at recruitment, and depending on the variable, related to current and/or past behaviours. For example, for smoking, questions included “Have you ever been a regular smoker?”, and if yes, “Are you a regular smoker now?” (Table 1); for alcohol consumption, the questions referred to current behaviour at recruitment: “On how many days each week do you usually drink alcohol?” and “About how many alcoholic drinks do you have each week?”; for reproductive behaviour, the questions related to past events: “How many children have you given birth to?”, “How old were you when you gave birth to your first child?”, and “How old were you when you gave birth to your last child?”. Extreme values were set to missing (Supplementary Table S1). All analyses were carried out separately for females and males. To check robustness, we randomly divided the data into equal sized discovery and validation datasets.

**Table 1:** Characteristics of the 45 and Up Study cohort at baseline, including age and all cancer risk variables used in the factor analysis.

Characteristic	Questionnaire item or definition	Females (n = 120,771)			Males (n = 100,891)		
		Missing post QC^$^	Mean (sd^$$^), or frequency	Median (IQR^^^)	Missing post QC^$^	Mean (sd^$$^), or frequency	Median (IQR^^^)
Age at baseline	Difference between baseline questionnaire date and date of birth	0	61 (11)	59 (52–68)	0	62 (11)	61 (54–70)
Smoking status	“Have you ever been a regular smoker?”, and if yes, “Are you a regular smoker now?”	38			43		
Never			78,365			49,518	
Former			34,088			43,213	
Current			8280			8117	
Years smoking regularly	Current smokers: difference between age at baseline and age from "How old were you when you started smoking regularly?"; former smokers: difference between age from "How old were you when you stopped smoking regularly?" and age from "How old were you when you started smoking regularly?"	2794	8 (14)	0 (0–12)	3458	13 (16)	0 (0–25)
Cigarettes/week	7 times number of cigarettes from "About how much do you/did you smoke on average each day?"	850	38 (65)	0 (0–70)	857	71 (96)	0 (0–140)
Alcohol drinks/week	“About how many alcoholic drinks do you have each week? One drink = one glass of wine, middy of beer, or nip of spirits”	2695	4 (6)	2 (0–7)	1498	10 (12)	6 (1–14)
Days of drinking alcohol/week	“On how many days each week do you usually drink alcohol?”	3071	2 (3)	1 (0–5)	1946	3 (3)	3 (1–6)
Red meat times/week	“About how many times each week do you eat beef, lamb, or pork?”	4329	3 (2)	3 (2–4)	3155	4 (3)	3 (2–5)
Processed meat times/week	“About how many times each week do you eat processed meat? Include bacon, sausages, salami, devon, burgers, etc.”	19,704	1 (1)	1 (0–2)	13,195	2 (2)	1 (1–2)
Fruit portions/week	7 times number from "About how many serves of fruit do you usually have each day?”	4401	14 (9)	14 (7–21)	4769	13 (10)	14 (7–14)
Cooked vegetables portions/week	7 times number in "About how many serves of raw vegetables do you usually eat each day?"	4770	19 (11)	21 (7–28)	3822	16 (11)	14 (7–21)
Raw vegetables portions/week	7 times number in "About how many serves of raw vegetables do you usually eat each day?"	12,530	12 (10)	7 (7–14)	15,556	10 (9)	7 (7–14)
Brown bread slices/week	“About how many slices or pieces of brown/wholemeal bread do you usually eat each week?”	6768	9 (7)	8 (4–14)	6030	12 (10)	10 (4–16)
Breakfast cereal bowls/week	“About how many bowls of cereal do you usually eat each week?”	9234	5 (3)	6 (2–7)	7065	5 (3)	6 (2–7)
Walking sessions in last week	“How many times did you walk continuously, for at least 10 min, last week?”	7777	5 (6)	4 (2–7)	6446	6 (7)	5 (2–7)
Walking hours in last week	“How much time did you spend altogether walking continuously, for at least 10 min, last week?”	10,395	3 (5)	2 (1–4)	7995	3 (5)	2 (1–4)
Moderate exercise sessions in last week	“How many times did you do moderate physical activity last week? (like gentle swimming, social tennis, vigorous gardening or work around the house)?”	11,867	4 (5)	3 (2–7)	9888	4 (6)	3 (1–6)
Moderate exercise hours in last week	“How much time did you spend altogether doing moderate physical activity last week? (like gentle swimming, social tennis, vigorous gardening or work around the house)?”	13,286	6 (9)	3 (1–7)	10,783	5 (8)	2 (1–6)
Vigorous exercise sessions in last week	“How many times did you do vigorous physical activity last week? (that made you breathe harder or puff and pant, like jogging, cycling, aerobics, competitive tennis, but not household chores or gardening)?”	22,343	1 (2)	0 (0–2)	16,061	2 (4)	1 (0–3)
Vigorous exercise hours in last week	“How much time did you spend altogether doing vigorous physical activity last week? (that made you breathe harder or puff and pant, like jogging, cycling, aerobics, competitive tennis, but not household chores or gardening)?”	23,055	1 (2)	0 (0–2)	16,881	1 (3)	0 (0–2)
Sleeping hours/week	7 times number in "How many hours in each 24 h day do you usually spend sleeping (including at night & naps)?"	5682	54 (8)	56 (49–56)	4790	54 (9)	56 (49–56)
Sitting hours/week	7 times number in "How many hours in each 24 h day do you usually spend sitting?"	11,670	37 (20)	35 (21–49)	7883	40 (22)	35 (28–56)
Watching TV or using computer hours/week	7 times number in "How many hours in each 24 h day do you usually spend watching television or using a computer?"	5437	29 (17)	28 (14–35)	4040	30 (17)	28 (18–35)
Standing hours/week	7 times number in "How many hours in each 24 h day do you usually spend standing?"	17,082	33 (23)	28 (14–49)	11,476	32 (22)	28 (14–49)
BMI	Weight in kg divided by squared height in meters	10,429	27 (5)	26 (23–29)	6381	27 (4)	27 (24–29)
Number of supplements taken (range: 0–5 from list)	Sum of boxes ticked from "Which of the following medications have you taken for most of the last 4 weeks?" [slightly paraphrased for splitting medications and supplements]	1	1 (1)	1 (0–2)	0	1 (1)	0 (0–1)
Number of medicines taken (range: 0–27 from list)	Sum of boxes ticked from "Which of the following vitamins or supplements have you taken for most of the last 4 weeks?" [slightly paraphrased for splitting medications and supplements]	1	2 (2)	1 (0–2)	0	1 (2)	1 (0–2)
Number of children born	“How many children have you given birth to?”	774	2 (1)	2 (2–3)			
Age when had first child	“How old were you when you gave birth to your first child?”	18,389	25 (5)	24 (21–28)			
Age when had last child	“How old were you when you gave birth to your last child?”	18,534	30 (5)	30 (27–34)			
Months breastfeeding	“For how many months, in total, have you breastfed?	2543	13 (15)	8 (1–18)			
Years used hormonal contraceptives	“Have you ever used the pill or other hormonal contraceptives?” and “If Yes, for how long altogether have you used hormonal contraceptives? [years]” [setting years used hormonal contraceptives to 0 if the answer to the first question was “no”]	5707	8 (8)	5 (0–11)			
Menopausal status	“Have you been through menopause?”	3411					
No			16,497				
Not sure (e.g. HRT/MHT)			15,513				
Irregular periods			8396				
Yes			76,954				
MHT use	"Have you ever used hormone replacement therapy (HRT)?" and if yes, "Are you currently taking HRT?"	2771					
Never			74,108				
Former			31,458				
Current			12,434				
Years used MHT	“How many years altogether have you used HRT?”	4174	3 (5)	0 (0–2)			
Enlarged prostate	“Has a doctor ever told you that you have an enlarged prostate?”				0		
No						86,830	
Yes						14,061	
Ability to get erection	“How often are you able to get and keep an erection that is firm enough for satisfactory sexual activity?”				3484		
Always						36,564	
Usually						22,677	
Sometimes						16,220	
Never						13,333	
*Rather not answer*						8613	
Leaking urine/week	“About how many times a week are you usually troubled by leaking urine?”				3210		
Never						80,884	
Once or less						9285	
2–3 times						4088	
4–6 times						1435	
Every day						1989	

^$^ *Missing post QC*  Missing values after exclusion of outliers (see Supplementary Table S1).

^$$^ *sd*  standard deviation.

^^^ *IQR*  interquartile range (25%-75%).

### Correlations between cancer risk variables

As some variables were continuous (e.g. BMI), others categorical (e.g. never/former/current smoker), pairwise correlations between all variables were calculated as polychoric correlations based on pairwise complete observations, using the Stata package polychoric^12,13^ (downloaded from http://www.komkon.org/~tacik/stata/).

### Identification of combined factors

To identify “combined factors” representing risk factor exposure patterns, we carried out a factor analysis based on the matrix of correlations between cancer risk variables, applying the Stata function “factormat”. Considering a scree plot, we retained 8 factors each for females and for males (Supplementary Fig. S1). We applied a varimax rotation to the 8 factors using the Stata function “rotate, varimax”. This yielded the “combined factors”. We found high agreement between the results from the discovery and validation datasets (Supplementary Note), and used the loadings from the discovery dataset in subsequent analyses.

#### Imputation of missing information

Missing data for cancer risk variables were imputed using a a nonparametric random forest method, applying the function “missForest” in the R package “missForest”^14^, with option variablewise = TRUE. Computation was parallelised by randomly splitting the discovery and validation datasets for males into 10 subsets each (9 subsets with 5000 individuals, plus remaining in subset 10). For females, the discovery and validation datasets were analogously split into 12 subsets each. Information was then imputed within each subset. This procedure was repeated 10 times, to yield 10 fully imputed datasets. We checked that imputation of the missing data did not change the mean or range of any variables.

#### Calculation of combined factor scores

For each fully imputed dataset, we calculated factor scores for all individuals using the function “factor.scores” in the R package “psych”^15^ with option method = “Thurstone”. This method calculates the regression based weights as W = R^−1^F, where R is the correlation matrix and F is the factor loading matrix^16^. The factor scores are then obtained as S = ZW, where Z is the matrix of standardised observed variables. For each participant and each combined factor, the score was calculated as the mean of the scores from the 10 imputations (Supplementary Fig. S2).

As there are different approaches for obtaining factor scores, each seeking to minimise a particular estimate of error, as a sensitivity analysis, we also calculated scores using the method = “Anderson” option. This method calculates weights such that the factor scores are uncorrelated as W = U^-2^F(F’U^-2^RU^-2^F)^-1/2^, where R and F are as defined above and U is the diagonal matrix of uniquenesses^16^. Based on the individual across-imputation mean scores, the correlations between the Thurstone and Anderson methods were extremely high (Pearson r 0.985–0.999), so scores based on the Thurstone method were used in subsequent analyses.

### Study sample for association analyses

Cancer incidence data for 2006–2013 were obtained from linkage to the NSW Cancer Registry (Supplementary Note, Fig. 1), using corresponding ICD-10-AM topological codes for all invasive cancers (C00-C96, D45-47.1,47.3–47.5), and for lung (C34), bowel (C18–C20), breast (C50), prostate cancer (C61), and melanoma (C53). The cancer incidence data included the month and year of diagnosis. To calculate the time between baseline questionnaire and cancer diagnosis, the day of diagnosis was set to 15. This resulted in 4–8 years of follow-up data (median 5.4 years, 25–75% range 5.3–5.9 years for 210,471 participants included in the association analysis for cancer incidence, see below and Fig. 1).

### Associations between combined factors and health, ancestry, and socioeconomic characteristics

We tested the association between each combined factor and age at baseline, as well as key health, ancestry, and socioeconomic characteristics (Supplementary Table S2). We used linear regression for each combined factor with all health, ancestry, and socioeconomic characteristics in a joint model. We defined significance at *P* < 0.001 to account for multiple testing (sensitivity analyses see Supplementary Note).

### Associations between combined factors and cancer incidence

We tested the association between each combined factor and cancer incidence (separately for all cancers, and for lung, bowel, breast, prostate cancers, and melanoma) using logistic regression. In each logistic regression analysis, cases were participants newly diagnosed with cancer after recruitment (separately for all cancers, lung, bowel, breast, and prostate cancer, and melanoma), while all other participants were included as non-cases. We applied the function “glm” in R with option family = “binomial” to estimate odds ratios (ORs) and the function “confint.default” to obtain 95% confidence intervals.

The covariates included were age, BMI, private health insurance, remoteness of residence index (ARIA)^17^, self-reported health rating, and number of GP visits in the 2 years prior to baseline (Supplementary Tables S2, S3). To capture GP visits, we used Medicare claims records and excluded 4759 female and 5778 male clients of the Australian Government’s Department of Veterans’ Affairs (DVA), as their healthcare is covered by a different billing system and may not be fully captured in the databases available for the 45 and Up Study cohort. DVA clients were identified through self-report in the 45 and Up Study baseline questionnaire, or through any mention of DVA coverage in a hospitalisation or emergency department presentation record. GP visits were identified using the MBS data (item codes 3–51).

We also adjusted for self-reported pre-baseline cancer screening: mammographic screening for breast and all cancers for females, prostate-specific antigen (PSA) testing for prostate and all cancers for males, and bowel screening for bowel and all cancers for males and females. For analyses of melanoma risk, we further adjusted for skin colour, tannability, and average daily hours outdoors.

We conducted two sensitivity analyses: testing all combined factors jointly; excluding all individuals with cancer diagnosed in the first year after the individual’s baseline questionnaire. Statistical significance was defined as *P* < 0.00125 in the main analysis (Bonferroni correction for 40 tests per gender), also requiring *P* < 0.05 in both sensitivity analyses.

We also verified that the estimates for the factor effects from logistic regression were not substantially different when additionally adjusted for highest educational qualification, income, and the relative socio-economic disadvantage index for areas (SEIFA, as calculated by the Australian Bureau of Statistics).

To further verify the results, we also carried out a survival analysis using competing risks regression for cancer incidence with death as the competing risk (“proportional sub-distribution hazards” regression model described by Fine and Gray^18^). As with the logistic regression approach, we tested each combined factor separately. In a sensitivity analysis, we also tested all combined factors jointly. Significance was defined as *P* < 0.00125 in the main analysis, with a further requirement of *P* < 0.05 in the sensitivity analysis. These analyses were done using the function “crr”^18^ in the R package “cmprsk”, with 95% confidence intervals for estimates obtained using the function “summary.crr”.

We note that competing risks regression has the advantage of explicitly taking into account follow-up time for individual participants, but the sub-distribution hazard includes individuals who have died in the risk set for cancer diagnosis^19^. This can cause difficulties in interpretation, hence logistic regression was presented as the main analysis, and all results were verified using competing risks regression.

#### Tests for interaction

Exposures to different cancer risk factors can have synergistic effects on cancer risk, for example, as found for smoking and alcohol for cancers of the upper aerodigestive tract^20^. Similar to comprehensive, non-hypothesis-driven assessments of individual risk factors, it is also of interest to examine potential interactions between pairs of risk factors to help identify areas for further investigation. However, large sample sizes are required for statistical interaction tests, and the multiple-testing correction required to systematically examine interactions can be prohibitive when examining many pairs of risk factors. Here, we leveraged the dimensionality reduction offered by the use of combined factors to test for interactions in a staged approach.

First, for cancer incidence, we tested interactions between combined factors using logistic regression as described above and including the interaction terms between pairs of combined factors. We only tested interactions between combined factors that were significantly associated with incidence of the same cancer type, and for that cancer type only (9 interactions for females, 3 for males; Supplementary Note).

Second, to further investigate an interaction between ‘alcohol’ and ‘menopausal hormone therapy (MHT)’ combined factors, we also tested for interactions between each of the two original alcohol variables with each of the two original MHT variables, using the same approach as for the combined factors. When analysing the original variables, we carried out tests based on the original data with exclusion of missing values. We verified that similar results were obtained when using across-imputation means from missForest imputation of missing data. Finally, we carried out a stratified analysis of breast cancer risk by baseline MHT status (never/former/current use) for all females and, separately, for post-menopausal females. In each stratum, we separately tested associations between breast cancer incidence and each of the ‘alcohol’ combined factor and both original alcohol variables.

## Results

### Correlations between cancer risk variables

We calculated pairwise correlations between 33 variables for females and 28 variables for males (Supplementary Table S4).

The highest correlations were observed between variables in the same domain (e.g. smoking behaviour: years smoked and number of cigarettes per week). We also observed correlations between smoking behaviour and consumption of alcohol (positive), fruit (negative), and breakfast cereal (negative). While most of these correlations were relatively weak, some of them were almost as strong as correlations between related variables such as fruit and vegetable consumption, or red meat and processed meat consumption.

Most correlations were similar for females and males (see Supplementary Note for description of differences).

### Identification of combined factors representing exposure patterns

**Figure 2:**
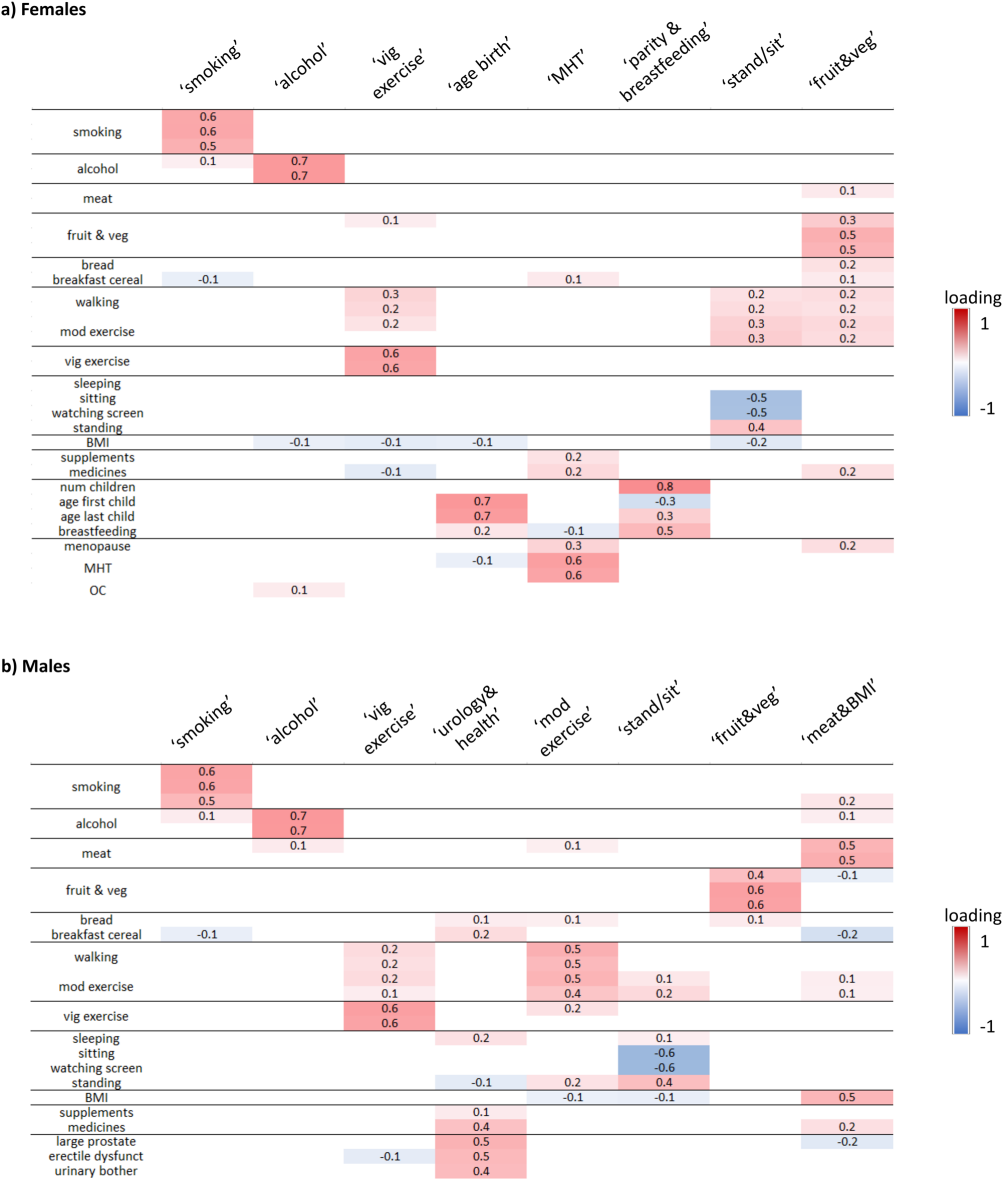
Combined factors for (**a**) females and (**b**) males. Original cancer risk variables shown in rows and combined factors in columns; combined factor labels reflect highest absolute loadings. For clearer visualisation only, loadings with absolute value ≥ 0.1 are shown and original variables are grouped into categories (full results: Supplementary Table S5). *vig* vigorous, *age birth*  age at childbirth, *MHT* menopausal hormone therapy, *stand/sit* standing/sitting, *veg* vegetables, *mod* moderate, *BMI* Body Mass Index, *OC* hormonal contraceptives, *dysfunct* dysfunction.

For females, factor analysis identified 8 “combined factors” that capture the variation in the original 33 variables and reflect exposure patterns. We labelled each combined factor based on the original risk variables with the strongest absolute loadings (Fig. 2a, Supplementary Table S5): ‘smoking’, ‘alcohol’, ‘vigorous exercise’, ‘age at childbirth’, ‘Menopausal Hormone Therapy (MHT)’, ‘parity & breastfeeding’, ‘standing/sitting’ (more time standing and less time sitting), and ‘fruit & vegetables’. We refer to the combined factors by their label as e.g. ‘smoking’ factor. We note that while the labels reflect the strongest absolute loadings, each factor also captured some information from other variables. For example, the ‘smoking’ factors for both females and males also captured some information on alcohol and breakfast cereal consumption.

Eight combined factors were also identified for males (Fig. 2b), labelled as ‘smoking’, ‘alcohol’, ‘vigorous exercise’, ‘urology & health’ (more urological symptoms and worse health), ‘moderate exercise’, ‘standing/sitting’, ‘fruit & vegetables’, and ‘meat & BMI’.

Combined factors with the same label for females and males may have different loading contributions from the original risk variables, due to differences in strengths of correlations. For example, for males, there was a stronger correlation between red meat and alcohol consumption, therefore a larger loading of red meat in the ‘alcohol’ combined factor (Fig. 2).

### Associations between combined factors and health, ancestry, and socioeconomic characteristics

**Figure 3:**
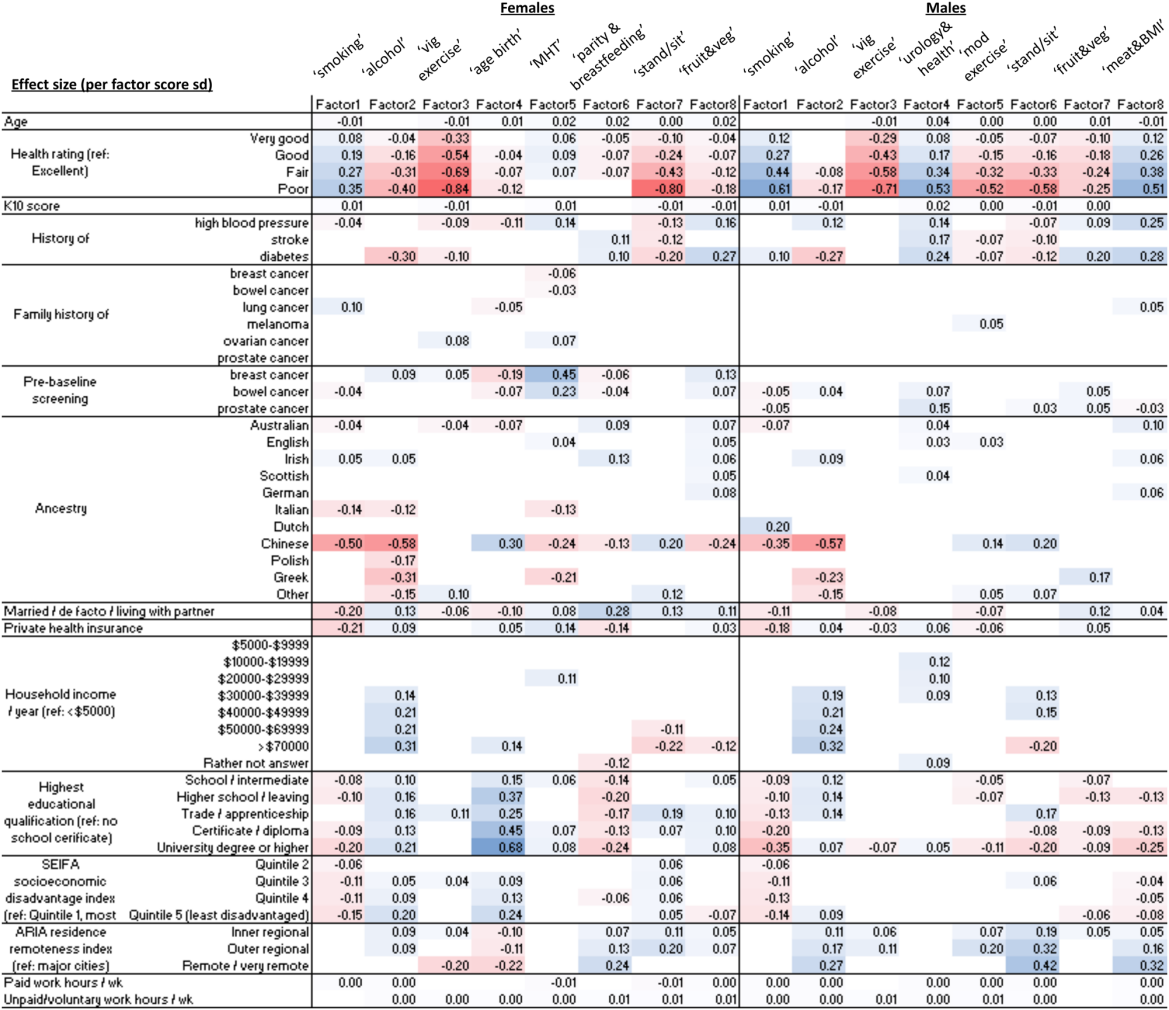
Associations between combined factors and sociodemographic characteristics for females and males. Figure shows coefficients from linear regression of each factor (in columns) jointly on all sociodemographic characteristics (in rows), where estimate has *P* < 0.001. Estimates shown are per unit of standard error for each factor. Blue: positive association, red: negative association. *sd* standard deviation; *vig* vigorous, *age birth* age at childbirth, *MHT* menopausal hormone therapy, *stand/sit* standing/sitting, *veg* vegetables, *mod* moderate, *BMI* Body Mass Index, */ wk* per week; pre-baseline screening row for prostate cancer  here refers to PSA testing.

The associations between each of the combined factors and age, ancestry, health, participation in cancer screening, family history of cancer, and socioeconomic characteristics are shown in Fig. 3.

We detected associations between the self-reported health rating and most of the combined factors, even when accounting for all other characteristics (i.e. age, ancestry, participation in cancer screening, family history of cancer, and socioeconomic characteristics). In particular, we identified associations between poorer health rating and higher ‘smoking’, ‘urology & health’ and ‘meat & BMI’ factor scores, as well as lower ‘alcohol’, ‘vigorous exercise’, ‘moderate exercise’, ‘age at childbirth’, ‘standing/sitting’, ‘fruit & vegetables’ factor scores.

We detected several associations with self-reported ancestry, including lower ‘smoking’ and ‘alcohol’ factor scores with Chinese ancestry; lower ‘smoking’ and higher ‘fruit & vegetables’ factor scores with Australian ancestry; higher ‘alcohol’ and, for females, higher ‘smoking’ factor scores with Irish ancestry; and lower ‘alcohol’ factor scores with Greek ancestry.

Married/de-facto/living-with-partner status was associated with lower ‘smoking’ and higher ‘fruit & vegetable’ factor scores, but also lower ‘vigorous exercise’ factor scores. For females, it was also associated with higher ‘alcohol’ and ‘parity & breastfeeding’ factor scores.

Characteristics reflecting higher socioeconomic advantage (e.g. private health insurance, higher education) were generally associated with lower ‘smoking’ and higher ‘alcohol’ factor scores, as well as higher ‘age at childbirth’ and lower ‘parity & breastfeeding’ factor scores for females.

### Associations between combined factors and cancer incidence

**Figure 4:**
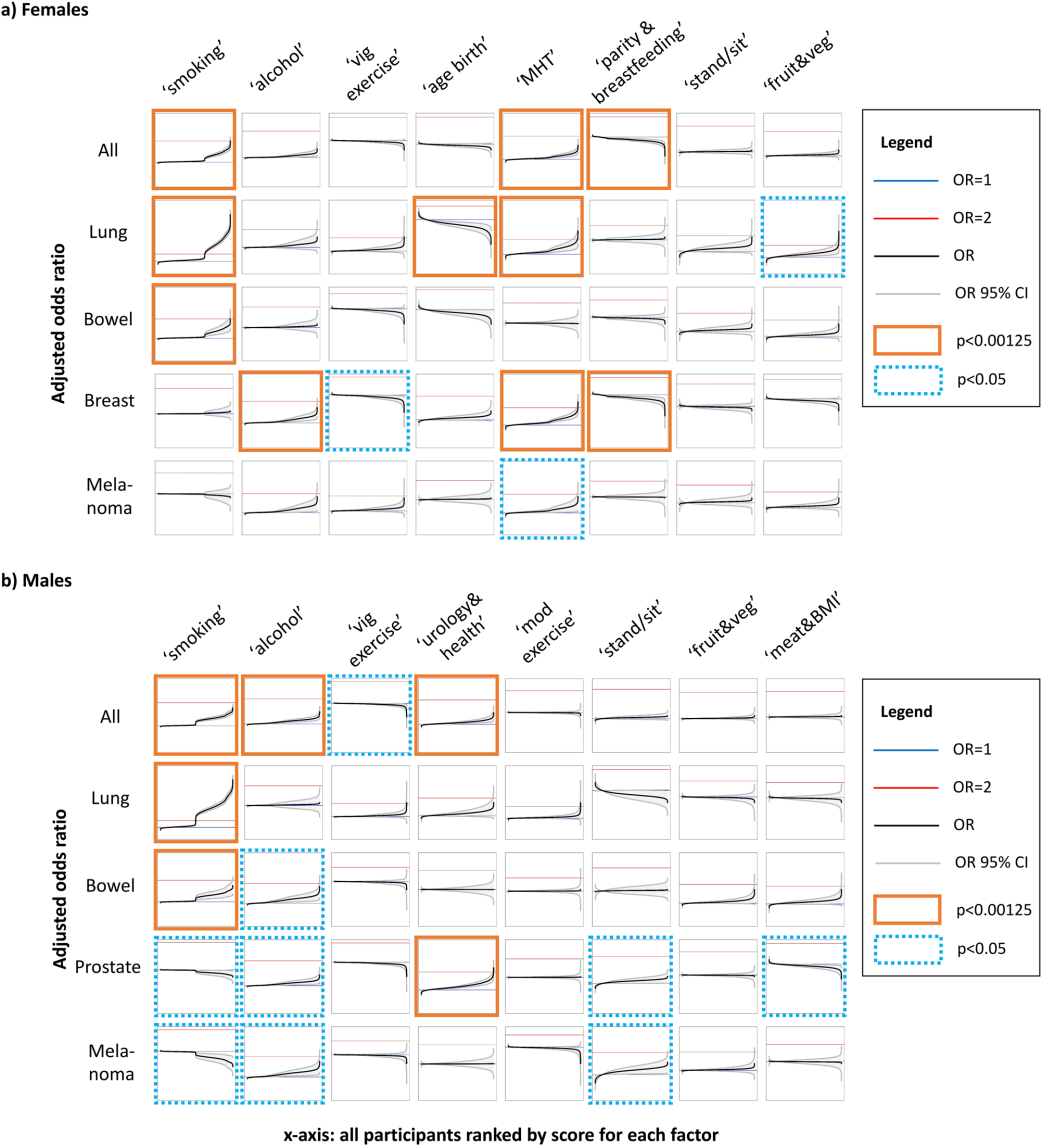
Association between combined factor scores and cancer incidence for (**a**) females and (**b**) males. Adjusted odds ratio (OR; y-axis) for study participants depending on factor score (x-axis), with all other covariates held constant, and the individual with 12.5% percentile score as reference (OR = 1). Odds ratios are adjusted for age, BMI, self-reported health at baseline, the number of GP visits in the 2 years prior to baseline, private health insurance, remoteness of residence, and where relevant, self-reported participation in cancer screening prior to baseline, or tannability-related covariates (see “Methods”). All estimates and results from sensitivity analyses see Supplementary Table S6. *vig* vigorous, *age birth* age at childbirth, *MHT* menopausal hormone therapy, *stand/sit* standing/sitting, *veg* vegetables, *mod* moderate, *BMI* Body Mass Index.

After correction for multiple testing and conducting several sensitivity analyses, we detected 10 associations between combined factors and cancer incidence for females and 6 for males (Fig. 4, Supplementary Table S6).

Of the 16 associations, 14 reflect well-known risk factors for cancer, such as the association of the ‘smoking’ factor with all cancers, lung cancer^21^, and bowel cancer^22^ for both females and males (Supplementary Note). There is conflicting or no prior evidence for the associations identified in this analysis between MHT use and lung cancer^23,24^ and age at childbirth and lung cancer, likely due to residual confounding (Supplementary Note).

Notably, some associations between combined factors and cancer incidence possibly reflect multiple mechanisms: for example, the factor ‘parity & breastfeeding’, which was associated with breast cancer incidence, has positive loadings for number of children, time breastfed, and negative loading for age at first childbirth, all of which are known to be associated with breast cancer^25,26^.

Several additional associations were only suggestive and did not pass multiple testing correction (3 for females, 9 for males). Several of these represent previously reported relationships (Supplementary Note).

### Interactions between combined factors associated with cancer risk

We found a possible interaction between ‘age at childbirth’ and ‘MHT’ factors for lung cancer incidence for females [adjusted odds ratio (OR) 1.17 (95% confidence interval (CI) 1.02–1.33); Supplementary Table S7]. However, we also found that smoking was higher among current than former and never MHT users at baseline (Supplementary Table S8). The interaction between ‘age at childbirth’ and ‘MHT’ factors was attenuated when also adjusting for the ‘smoking’ factor. Hence this interaction was not investigated further.

We also found a possible interaction effect between ‘alcohol’ and ‘MHT’ factors for breast cancer incidence [adjusted OR 1.06 (95% CI 1.00–1.12), p = 0.046; Supplementary Table S7]. To follow up this result and appropriately consider menopausal status, we focused on females post-menopause at baseline, and stratified them by never/former/current MHT use at baseline. The association with the ‘alcohol’ factor was strongest for current MHT users (Table 2), with similar results when using the original variables of weekly alcohol drinks and days drinking alcohol. Unfortunately, data on MHT type were not available to stratify the cohort further, and it is known that the association between MHT and breast cancer incidence varies substantially by MHT type^27^. Moreover, current MHT users also reported higher alcohol intake, and the confidence intervals for odds ratios overlapped between strata, hence these results are interpreted as suggestive only.

**Table 2:** Association between alcohol and breast cancer incidence, stratified by MHT use, with a focus on post-menopausal females to adequately reflect dependencies between MHT use and menopausal status.

	Adjusted^a^ OR (95% CI)^b^		
	Never MHT	Current MHT	Former MHT
**All females (n = 115,715)**			
‘Alcohol’ combined factor	1.05 (0.97–1.13)	1.17 (1.03–1.34) *	1.08 (0.98–1.19)
Days of drinking alcohol/week	1.02 (0.99–1.04)	1.06 (1.01–1.12) *	1.04 (1.00–1.07) *
Alcohol drinks/week	1.01 (1.00–1.02)	1.02 (1.00–1.04) *	1.01 (0.99–1.02)
BMI (in stratified analysis of alcohol factor)	1.03 (1.01–1.04) **	1.00 (0.97–1.03)	1.03 (1.01–1.04) **
**Post-menopausal females (n = 73,238)**			
‘Alcohol’ combined factor	1.04 (0.94–1.14)	1.27 (1.08–1.49) **	1.08 (0.97–1.21)
Days of drinking alcohol/week	1.01 (0.98–1.05)	1.09 (1.03–1.16) **	1.04 (1.00–1.08)
Alcohol drinks/week	1.01 (0.99–1.02)	1.03 (1.01–1.06) **	1.01 (0.99–1.02)
BMI (in stratified analysis of alcohol factor)	1.03 (1.01–1.04) **	1.00 (0.96–1.04)	1.03 (1.00–1.05) *

*CI* confidence interval.

^a^Adjusted for age, Body Mass Index (BMI), self-reported health at baseline, the number of GP visits in the 2 years prior to baseline, self-reported participation in breast screening prior to baseline, private health insurance, remoteness of residence.

*P < 0.05, **P < 0.01.

^b^OR = odds ratio (per 1 unit change in the continuous variable).

Since previous studies reported interactions between MHT use and BMI^28–30^, we examined the association between BMI and breast cancer risk stratified by MHT status (Table 2). BMI was associated with breast cancer incidence for never MHT users and former MHT users, but not for current MHT users at baseline, as also reported previously^28^.

## Discussion

We have systematically examined the pairwise correlations between 36 cancer risk variables for over 220,000 Australian residents, and identified 8 “combined factors” each for females and for males, which capture exposure patterns. We detected extensive associations between the combined factors and sociodemographic characteristics such as self-rated health, medical history, family history of cancer, participation in cancer screening, ancestry, private health insurance, income, education, area-based socio-economic disadvantage, and remoteness of residence. We also identified 16 significant associations between the combined factors and cancer incidence, of which 14 represent well-known relationships, providing evidence for the validity of this approach.

The comprehensive characterisation of correlations between over 30 cancer risk exposures (and thus their degree of co-dependency) in this study has a range of important applications, from studies of cancer risk, to microsimulation modelling and the design of interventions.

Correlation between cancer risk exposures can lead to confounding in studies of cancer incidence, leading to e.g. possibly spurious associations between smoking and breast cancer due to confounding by alcohol consumption^31^. For future studies focused on specific single exposures, the correlations with other exposures provided in this study will allow better identification and examination of possible confounders. Similarly, the atlas of associations between combined factors and sociodemographic characteristics can also help to identify possible confounders for future studies of cancer risk.

Knowledge of relationships between risk factor exposures is also crucial for microsimulation modelling, which simulates millions of individuals in a population to forecast future disease burden and the effects of interventions. For cancer risk, current models typically only simulate an overall underlying cancer risk, e.g.^32–34^ or only one risk factor^35,36^. The next step would be to create more holistic models with realistic constellations of multiple exposures, such as both smoking and alcohol intake for bowel cancer. This again requires information on correlations between these exposures, such as provided by this study.

In another key area of application, information on relationships between risk factor exposures also underlies the development of comprehensive intervention programmes that help people modify their lifestyles. While targeting multiple, possibly uncorrelated behaviours simultaneously can reduce the completion rate of interventions^37^, targeting correlated behaviours might improve success. For example, one study found that a joint intervention for smoking and alcohol intake temporarily reduced smoking better than an intervention for smoking alone^38^, and that smoking lapses often occurred with alcohol use^39^. Moreover, the atlas of associations between cancer-relevant risk behaviours and sociodemographic characteristics provides information for the design of targeted intervention approaches to include social determinants, suggesting which population groups have higher exposure to given risk factors. For example, we found that remoteness of residence was associated with both higher ‘alcohol’ and ‘meat & BMI’ combined factor scores for males, suggesting potential interventions to reduce alcohol intake, meat consumption, or obesity levels might be targeted to remote regions.

In addition to dependencies between cancer risk factor exposures, it is possible that the effects of some exposures on cancer risk may not be independent. Very large sample sizes are necessary to reliably detect interactions, hence the results in this study are provided to generate hypotheses for testing in future work. We found a possible interaction between alcohol consumption and MHT status on breast cancer risk, with the highest risk for alcohol consumption for females taking MHT at recruitment (i.e. a departure from a multiplicative model). Alcohol is known to increase breast cancer risk for both pre- and post-menopausal females, with likely complex causal mechanisms^40^. Previous meta-analyses have shown that alcohol consumption affects sex hormone levels including oestradiol^41^, and the increase in circulating oestradiol levels with alcohol consumption is thought to affect the formation or growth of cancerous cells^42^. Notably, a small double-blind, placebo-controlled crossover study found that alcohol consumption led to a threefold increase in circulating oestradiol for females taking MHT, with no significant change in those not taking MHT^43^. However, residual confounding remains a possibility. Hence larger follow-up studies will be crucial to confirm whether an interaction effect is present, and if so, whether it relates to a specific MHT type.

Some of the associations identified between combined factors and cancer incidence can also serve to generate new hypotheses to be followed up in more targeted studies. As expected and noted above, almost all (14/16) of the most significant associations reflect well-known cancer risk factors (Supplementary Note). Of the nominally significant associations (0.00125 < *P* < 0.05), several reflect relationships that have also been reported previously, including associations between the ‘vigorous exercise’ factor and breast cancer^44^ incidence for females and incidence of all cancers^45^ for males (decreasing risks with higher scores), and between the ‘alcohol’ factor and bowel^46^ and prostate cancer^47^ incidence for males (increasing risks with higher scores). Some associations have contradictory evidence from past studies and should thus be considered as potential false-positives due to chance or confounding. For example, some cohort studies have also reported increased melanoma incidence with MHT use (e.g. specifically for estrogens^48^), although a small clinical trial did not find a significant effect^49^. It is possible that the association depends on MHT type, data for which were not available in this study.

This study has several limitations. First, the 45 and Up Study participants were limited to those aged at least 45 years. While we did not see different correlations between original risk variables by 10-year age groups (data not shown), these correlations cannot necessarily be generalised to those below 45 years of age. The generalisability is also limited by sampling bias of participants, who are known to be healthier and of lower social disadvantage than the general population^9^. Moreover, the correlations may be different among specific population subgroups (e.g. by social disadvantage, or cultural background); investigating this was beyond the remit of this study. We also note that the correlations between risk factor exposures and the associations between risk factor exposure patterns and sociodemographic characteristics may be different in other countries. However, representativeness is not required for reliable relative risk estimates from internal comparisons, e.g. when testing associations between combined factors and cancer incidence^50^. Second, the data on cancer risk exposures and sociodemographic characteristics were self-reported, which could lead to biases due to participants’ recall. While past work has shown that e.g. self-reported use of medications for chronic conditions agreed well with administrative data^51^, this might not extend to lifestyle behaviours, especially exposures or characteristics that are possibly stigmatised. Moreover, for some risk behaviours, the question related to usual behaviour around the time of recruitment (e.g. “On how many days each week do you usually drink alcohol?”). Thus, information on cumulative lifetime risk exposure was only available for some of the risk factors. Third, this study was limited to available data, for example, it is known that cancer risk differs by MHT type^52^, but this information was not available. Fourth, we used the number of GP visits in the 2 years prior to baseline as a covariate in the analyses of cancer risk. As data to capture GP visits was only available from June 2004, this variable would not be captured correctly for the approximately 14% of participants who were recruited prior to June 2006. However, the second covariate used for health at recruitment (self-rated health) was captured for everyone. Finally, while it would be of interest to identify the exact contributions of the original exposure variables to the associations between combined factors and cancer incidence, these in-depth follow-up analyses are beyond the scope of the current study.

In summary, this study provides a large-scale, systematic analysis of cancer risk exposures in a large-scale population cohort. The identified relationships between risk variables can be used to inform a wide variety of future studies, and design interventions targeting multiple correlated behaviours. Further information for targeting such approaches is provided by the associations between combined factors and sociodemographic characteristics. This study also shows the potential of factor analysis as an approach for identifying associations between exposure patterns and cancer risk.

## Supplementary Information

Supplementary Information 1.

Supplementary Information 2.

## Data Availability

Access to the 45 and Up Study dataset was provided by the Sax Institute. MBS and PBS data from Services Australia were linked by the Sax Institute. NSW Health data were provided by the NSW Ministry of Health and Cancer Institute NSW and probabilistically linked by the Centre for Health Record Linkage in NSW (CHeReL). Access procedures for the 45 and Up Study data are provided at https://www.saxinstitute.org.au/our-work/45-up-study/for-researchers. Generally, access is available to any bona fide researcher who: has a scientifically sound and feasible research proposal; has ethics approval for the proposal and data custodian approval for access to linked data, if required for the project; can meet 45 and Up Study licence and SURE user charges. Data access enquiries can be made to the Sax Institute (see https://www.saxinstitute.org.au/our-work/45-up-study/governance/ for details).
