# Supplementary material for: Large-scale systematic analysis of exposure to multiple cancer risk factors and the associations between exposure patterns and cancer incidence
Source: Sci Rep. 2021 Jan 27;11:2343. doi: 10.1038/s41598-021-81463-6 (PMC7841154; doi:10.1038/s41598-021-81463-6)
Supplement: Supplementary file 1 — Supplementary Information 1. [file 41598_2021_81463_MOESM1_ESM.pdf]

Large-scale systematic analysis of exposure to multiple cancer risk factors and the associations between exposure patterns and cancer incidence

Julia Steinberg[1,2,*], Sarsha Yap[1], David Goldsbury[1], Visalini Nair-Shalliker[1,2], Emily Banks[3], Karen Canfell[1,2,4], Dianne L. O'Connell[1,2,5]

[1] Cancer Research Division, Cancer Council NSW, Woolloomooloo, NSW, Australia

[2] Sydney School of Public Health, University of Sydney, Sydney, NSW, Australia

[3] National Centre for Epidemiology and Population Health, Australian National University, Canberra, Australia

[4] Prince of Wales Clinical School, UNSW Medicine, Sydney, NSW, Australia

[5] School of Medicine and Public Health, University of Newcastle, Newcastle, NSW, Australia

* Corresponding author: Julia.steinberg@nswcc.org.au

Supplementary Information

Contents

Supplementary Note ... 2

Correlations between cancer risk variables ... 2

Identification of combined factors ... 2

Associations between combined factors and health, ancestry, and socioeconomic characteristics 2

Associations between combined factors and cancer incidence ... 2

Supplementary Tables ... 5

Supplementary Figures ... 6

References ... 8

Supplementary Note

Correlations between cancer risk variables

For common risk variables, most correlations are similar for females and males (average absolute difference 0.03). The highest absolute difference is seen for the correlation between number of times per week doing moderate and vigorous exercise (r=0.18 females, r=0.33 males), followed by BMI and alcohol drinks per week (r=-0.11 females, r=0.03 males). Other strong differences include correlations between alcohol drinks per week and days drinking alcohol (r=0.81 females, r=0.69 males), as well as between red meat portions per week and alcohol drinks per week (r=0.05 females, r=0.13 males).

Identification of combined factors

To check for robustness, we carried out the factor analysis separately for discovery and validation datasets, with very high agreement between the results (Supplementary Fig. S1, Supplementary Table S5, Supplementary Table S9). Re-scaling rotated factors to a variance of 1 each, the mean difference in loadings was 0.005 for females (sd 0.005) and 0.01 for males (sd 0.01).

We note that the identification of combined factors is driven by correlations between exposure variables only. Thus, it is not influenced by the strength of associations between specific risk factors and cancer incidence.

Associations between combined factors and health, ancestry, and socioeconomic characteristics

We compared three approaches to test for associations.

In the first approach, for each factor we tested the associations of the across-imputation mean scores with the variables. In the second approach, we calculated the association of each factor with each variable separately in each imputed dataset, then combined information across the 10 imputations using Rubin's rule [1]. In the third approach, any missing values of original risk factors were substituted with the median prior to calculation of factor scores.

Defining significance at $P<0.001$, we found that the results were highly similar across these approaches. Mostly, the significance of associations was slightly attenuated when substituting any missing original risk factor values with the median, with highly similar effect size estimates. Consequently, we used the first approach (averaging individual factor scores across missForest imputations) in further analyses.

While factor scores were available for all individuals, only those without missing health, ancestry, and socioeconomic characteristics could be included in this analysis.

Associations between combined factors and cancer incidence

In the competing risks regression, as well as the sensitivity analyses excluding cancers diagnosed in the first year, we also excluded further 3 females and 3 males with possibly incorrect linkage to death records (death before baseline date).

The results for logistic regression and competing risks regression approaches were very similar (Supplementary Table S6), with e.g. the relative difference between OR and SHR estimates being <2% for significant associations ($P<0.00125$) in the logistic regression analysis. This is in line with past work finding that logistic regression models yield very similar results to those from proportional

hazards models, especially for rare outcomes, moderate risks, and follow-up periods under 10 years [2-4].
Thus, to increase interpretability, the results from the logistic regression are presented in the main paper.

We note that the association analyses of combined factor scores with cancer incidence were carried out for individuals without missing covariate information. For the competing risks regression, individuals for whom death occurred as the first event in the follow-up period were more likely to be excluded due to missing data (Supplementary Table S10), with little or no difference between individuals without any event and those with a cancer diagnosis. However, the proportion of participants who died during follow-up and before cancer diagnosis was small, and as noted above, there was strong agreement between the logistic regression and the competing risks regression.

Of the 16 significant associations after Bonferroni correction (Fig. 4), 14 reflect well-known cancer risk factors: association between the 'smoking' factor and all cancer, lung cancer [5], and bowel cancer [6] incidence for both females and males; associations between the 'alcohol' factor and breast cancer [7] incidence for females and all cancer incidence for males; association between the 'MHT' factor and all cancers and breast cancer [8] incidence for females; association between 'parity & breastfeeding' factor and all cancer and breast cancer [9] incidence for females; and associations between the 'urology & health' factor and all cancer and prostate cancer incidence for males.

Two of the significant associations have conflicting or no external evidence. A meta-analysis has found decreased lung cancer risk with MHT use [10], contrary to the significant association in this study, so the association here might be a false positive. The association between the 'age at childbirth' factor and lung cancer for females (decreasing risk with higher age at birth) could be due to residual confounding by socioeconomic status, and thus also lower exposure to second-hand smoke.

Of the nominally significant associations ($0.00125<P<0.05$), several reflect relationships that have also been previously reported, including associations between the 'vigorous exercise' factor and breast cancer [11] incidence for females and all cancers [12] incidence for males (decreasing risks with higher scores), and between the 'alcohol' factor and bowel [13] and prostate cancer [14] incidence for males (increasing risks with higher scores).

Other associations have contradictory evidence from past studies. For example, some cohort studies have also reported increased melanoma incidence with MHT use (e.g. specifically for estrogens [15]), although a small clinical trial did not find a significant effect [16]. It is possible that the association depends on MHT type, data for which were not available in this study. We accounted for more relevant covariates than past studies (skin colour, tannability, and outdoors hours per day), though not for immediate tanning behaviour (e.g. sunscreen use), so it is possible that deliberate sun-seeking could confounds this association. We also identified some associations with unclear mechanisms (e.g. for males, increased melanoma risk with higher 'alcohol' factor scores for males).

Some other associations are likely due to residual confounding. For example, the association between the 'smoking' factor and prostate cancer (decreasing risk) could be due to confounding by screening behaviour (less likely screening participation with higher smoking factor scores, Fig. 3). While we adjusted for binary self-reported pre-baseline PSA testing, residual confounding could be due to quantitative differences in testing frequency. Similarly, higher scores on the 'standing/sitting' factor are associated with higher likelihood of PSA screening (Fig. 3), so additional differences in testing frequency could give rise to the nominally significant association with prostate cancer incidence (Fig. 4). Moreover, the association between the 'meat & BMI' factor and prostate cancer incidence (decreasing risk) does not reflect a well-known relationship, as meat consumption is not known to be associated with prostate cancer, and BMI is thought to probably increase risk for advanced prostate

cancer [17] (opposite direction to the association observed here). This association could therefore also be a false positive.

We also note that the absence of a significant association could be due to lack of power. For example, for the ‘meat & BMI’ combined factor for males, the odds ratio estimate >1 for bowel cancer indicates potential increasing bowel cancer risk with increasing meat consumption and BMI, but the association does not reach statistical significance (*P*=0.09 in the main analysis).

Tests for interaction

We tested for pairwise interactions between all variables that were associated with incidence for each specific cancer type. This included testing for ‘smoking’, ’MHT’, and ’parity & breastfeeding’ factors with all cancer incidence for females; between ‘smoking’, ‘age at childbirth’, and ’MHT’ factors with lung cancer incidence for females; between ‘alcohol’, ’MHT’ and ‘parity & breastfeeding’ factors with breast cancer incidence for females; and between ‘smoking’, ‘alcohol’, and ‘urology & health’ factors with all cancer incidence for males (Supplementary Table S7).

Supplementary Tables

Supplementary Table S1. Cut-off thresholds for variables used in quality control of the data; values outside of the thresholds were set to missing.

Supplementary Table S2. Ancestry, health and socioeconomic characteristics of the 45 and Up Study cohort, restricted to participants with follow-up data.

Supplementary Table S3. Additional characteristics of the 45 and Up Study cohort used as covariates in the analysis of associations between combined factors and cancer incidence.

Supplementary Table S4. Polychoric correlations between cancer risk factors in the discovery and validation datasets, separately for females and males.

Supplementary Table S5. Loadings of the original risk variables onto the combined factors (re-scored to variance=1 for each factor).

Supplementary Table S6. Association of combined factors with cancer incidence in the main and sensitivity analyses using logistic regression, as well as in the competing risks regression analysis.

Supplementary Table S7. Interactions between combined factors on cancer incidence, including follow-up analyses for breast cancer for females using original alcohol consumption and MHT use variables.

Supplementary Table S8. Percentage of never smokers, mean number of years smoked and mean average number of cigarettes per week by MHT use.

Supplementary Table S9. Results of the factor analysis based on the polychoric correlations from Supplementary Table S6, separately for discovery and validation datasets for females, and discovery and validation datasets for males. Values shown are prior to varimax rotation.

Supplementary Table S10. Number of incident cancers and deaths before cancer diagnosis in the 45 and Up Study cohort.

Supplementary Figures

Supplementary Figure S1. Eigenvalue plot ("scree plot") for the factor analysis results for a) females and b) males, showing a close correspondence of results between the discovery and validation datasets. As the correlation matrix is not a true statistical correlation matrix, we note that some eigenvalues are negative, also leading to negative estimates for the proportion of variance explained for some factors (see Supplementary Table S9).

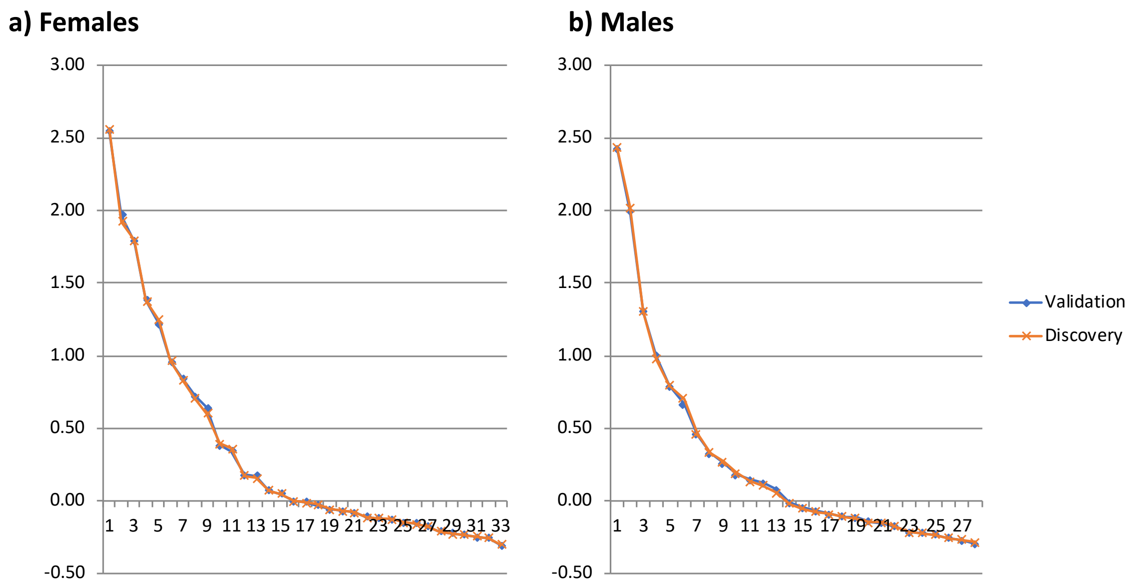

Supplementary Figure S2. Shape of the distribution of factor scores for a) females and b) males, for each of the 8 factors. The red vertical lines show the minimum, 20% percentile, 40% percentile, 60% percentile, 80% percentile, and maximum values (left to right).

a) Females

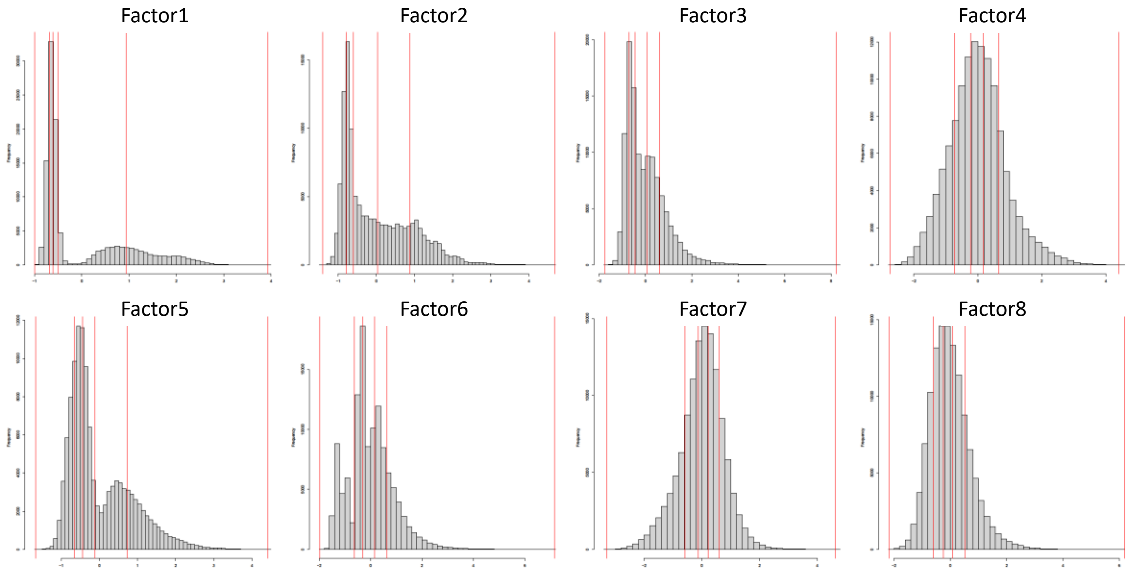

b) Males

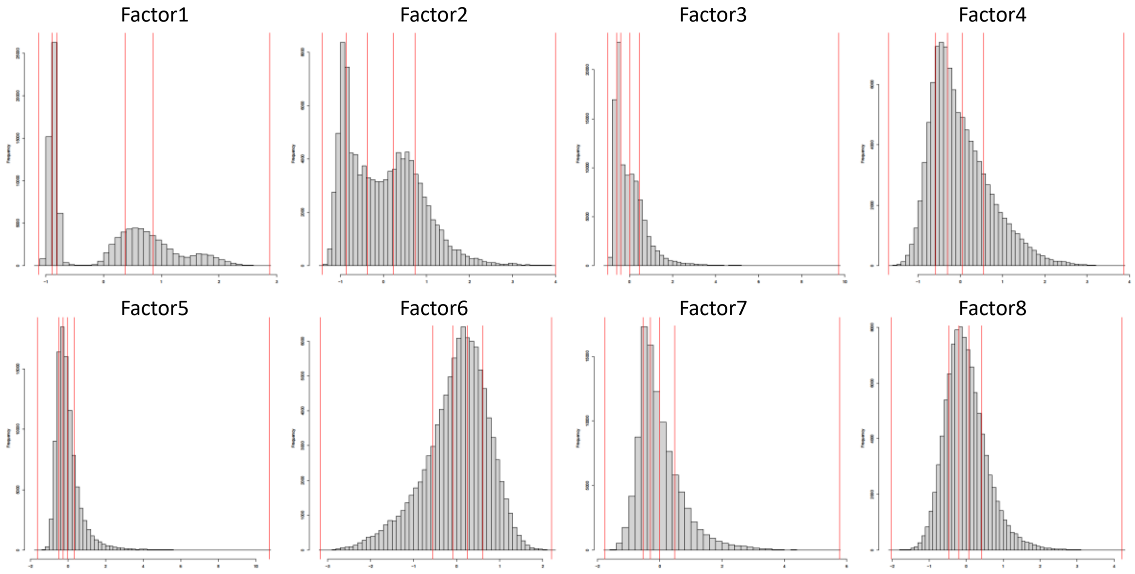

References

1. Rubin, D.B. Multiple Imputation for Nonresponse in Surveys. 2004, John Wiley and Sons: New York.
2. Ingram, D.D. and J.C. Kleinman. Empirical comparisons of proportional hazards and logistic regression models. *Stat Med*, 1989. **8**(5): p. 525-38.
3. Annesi, I., T. Moreau, and J. Lellouch. Efficiency of the logistic regression and Cox proportional hazards models in longitudinal studies. *Stat Med*, 1989. **8**(12): p. 1515-21.
4. Callas, P.W., H. Pastides, and D.W. Hosmer. Empirical comparisons of proportional hazards, poisson, and logistic regression modeling of occupational cohort data. *Am J Ind Med*, 1998. **33**(1): p. 33-47.
5. O'Keeffe, L.M., G. Taylor, R.R. Huxley, P. Mitchell, M. Woodward, et al. Smoking as a risk factor for lung cancer in women and men: a systematic review and meta-analysis. *BMJ Open*, 2018. **8**(10): p. e021611.
6. Botteri, E., S. Iodice, V. Bagnardi, S. Raimondi, A.B. Lowenfels, et al. Smoking and colorectal cancer: a meta-analysis. *JAMA*, 2008. **300**(23): p. 2765-78.
7. Bagnardi, V., M. Rota, E. Botteri, I. Tramacere, F. Islami, et al. Alcohol consumption and site-specific cancer risk: a comprehensive dose-response meta-analysis. *Br J Cancer*, 2015. **112**(3): p. 580-93.
8. Narod, S.A. Hormone replacement therapy and the risk of breast cancer. *Nat Rev Clin Oncol*, 2011. **8**(11): p. 669-76.
9. Collaborative Group on Hormonal Factors in Breast Cancer. Breast cancer and breastfeeding: collaborative reanalysis of individual data from 47 epidemiological studies in 30 countries, including 50302 women with breast cancer and 96973 women without the disease. *Lancet*, 2002. **360**(9328): p. 187-95.
10. Yao, Y., X. Gu, J. Zhu, D. Yuan, and Y. Song. Hormone replacement therapy in females can decrease the risk of lung cancer: a meta-analysis. *PLoS One*, 2013. **8**(8): p. e71236.
11. Hardefeldt, P.J., R. Penninkilampi, S. Edirimanne, and G.D. Eslick. Physical Activity and Weight Loss Reduce the Risk of Breast Cancer: A Meta-analysis of 139 Prospective and Retrospective Studies. *Clin Breast Cancer*, 2018. **18**(4): p. e601-e612.
12. Kerr, J., C. Anderson, and S.M. Lippman. Physical activity, sedentary behaviour, diet, and cancer: an update and emerging new evidence. *Lancet Oncol*, 2017. **18**(8): p. e457-e471.
13. Vieira, A.R., L. Abar, D.S.M. Chan, S. Vingeliene, E. Polemiti, et al. Foods and beverages and colorectal cancer risk: a systematic review and meta-analysis of cohort studies, an update of the evidence of the WCRF-AICR Continuous Update Project. *Ann Oncol*, 2017. **28**(8): p. 1788-1802.
14. Zhao, J., T. Stockwell, A. Roemer, and T. Chikritzhs. Is alcohol consumption a risk factor for prostate cancer? A systematic review and meta-analysis. *BMC Cancer*, 2016. **16**(1): p. 845.
15. Botteri, E., N.C. Stoer, S. Sakshaug, S. Graff-Iversen, S. Vangen, et al. Menopausal hormone therapy and risk of melanoma: Do estrogens and progestins have a different role? *Int J Cancer*, 2017. **141**(9): p. 1763-1770.
16. Tang, J.Y., K.M. Spaunhurst, R.T. Chlebowski, J. Wactawski-Wende, E. Keiser, et al. Menopausal hormone therapy and risks of melanoma and nonmelanoma skin cancers: women's health initiative randomized trials. *J Natl Cancer Inst*, 2011. **103**(19): p. 1469-75.
17. World Cancer Research Fund/American Institute for Cancer Research. Continuous Update Project Report 2018. Body fatness and weight gain and the risk of cancer.
